# Human Cytomegalovirus-Encoded microRNAs Can Be Found in Saliva Samples from Renal Transplant Recipients

**DOI:** 10.3390/ncrna6040050

**Published:** 2020-12-18

**Authors:** Shelley Waters, Silvia Lee, Kylie Munyard, Ashley Irish, Patricia Price, Bing H. Wang

**Affiliations:** 1 School of Pharmacy & Biomedical Science, Curtin Health Innovation Research Institute, Curtin University, Perth 6102, Australia; shelley.waters@postgrad.curtin.edu.au (S.W.); k.munyard@exchange.curtin.edu.au (K.M.); 2 Biomarker Discovery Laboratory, Baker Heart and Diabetes Institute, Melbourne 3004, Australia; bing.wang@baker.edu.au; 3 Department of Microbiology, Pathwest Laboratory Medicine, Murdoch 6150, Australia; silvia.lee@curtin.edu.au; 4 Renal Unit, Fiona Stanley Hospital, Murdoch 6150, Australia; ashley.irish@health.wa.gov.au

**Keywords:** cytomegalovirus, HCMV, kidney transplant, miRNA, saliva

## Abstract

Human cytomegalovirus (HCMV) infections are common following renal transplantation and may have long-lasting effects. HCMV can be measured directly by viral DNA or indirectly via host immune responses. HCMV-encoded microRNA (miRNA) may alter the pathobiology of HCMV infections and contribute to the progression of HCMV disease. HCMV-encoded miRNAs can be detected in blood but have not been sought in saliva. We investigated saliva samples from 32 renal transplant recipients (RTR) and 12 seropositive healthy controls for whom immunological data was available. Five HCMV-encoded miRNAs (miR-UL112-5p, miR-US5-2-3p, miR-UL36, miR-US25-2-3p and miR-UL22A) were sought using primer probe assays. HCMV miRNA species were detected in saliva from 15 RTR and 3 healthy controls, with miR-US5-2-3p most commonly detected. The presence of HCMV miRNAs associated with increased T-cell responses to HCMV IE-1 in RTR, suggesting a link with frequent reactivations of HCMV.

## 1. Introduction

Despite prophylactic measures, human cytomegalovirus (HCMV) remains a significant pathogen following renal transplantation, where infections contribute to end-organ disease, graft rejection and secondary bacterial and fungal infections [1]. HCMV is routinely monitored by the presence of viral DNA. However, HCMV does not replicate in blood leukocytes, so its DNA is often undetectable in blood or plasma [2]. Acinar cells of the salivary gland support HCMV replication and may be a prominent site of HCMV latency. Saliva is easily collected as a non-invasive sample, but the optimal assays and clinical utility of finding HCMV DNA in saliva are unclear.

The presence of HCMV in a host has also been assessed using antibody and T-cell responses, even though these are influenced by the host’s immunological capacity—notably in human immunodeficiency virus (HIV) patients [3]. Levels of IgG antibodies reactive with HCMV antigens reflect a lifetime history of infection and the cumulative viral burden. In older HCMV seropositive adults, up to 23% of the entire CD8^+^ T-cell compartment can be specific for HCMV [4]. HCMV-reactive antibodies also rise with age, and higher titres are associated with all-cause mortality, development of cardiovascular diseases and reduced responses to influenza vaccination in elderly populations [5].

Following organ transplantation, immunosuppressive regimes increase the frequency of HCMV reactivation and infection. Accordingly, renal transplant recipients (RTR) have higher levels of circulating antibodies than the general population [6]. Interferon γ-producing T-cells, particularly those specific for HCMV Immediate Early 1 (IE-1) antigen, are also increased in RTR [7]. Responses to this antigen are also elevated in HIV patients [8] and are thought to reflect frequent reactivations of HCMV as the antigen is produced in the earliest stages of the replicative cycle [9].

Human microRNAs (miRNAs) are implicated in mechanisms involved in establishing HIV latency [10]. Whilst relatively less abundant, HCMV encodes miRNAs itself. These have been described in vitro and detected in plasma or serum [11]. In addition to a role in monitoring infection, miRNA may shed light on pathogenesis—for example, by targeting host genes involved in immunomodulation, such as *MICB,* which modulates natural killer (NK) cell cytotoxicity [12]. Cytomegalovirus (CMV) encoded miRNAs have been identified in saliva from infected rhesus macaques (RhCMV) [13] and rats (RCMV) [14], but there are genomic differences between CMV strains infecting different species, and the existence of HCMV-encoded miRNA in saliva has not been addressed. Before consideration of HCMV-encoded miRNA for clinical use, we need to how and where they are expressed, which metrics of HCMV they associate with and which miRNA/s are most informative. Parallel in vitro studies (e.g., [15]) can then establish how key miRNA impact the hosts’ immune system. We have linked the detection of HCMV DNA in saliva with a systemic response to HCMV [16]. Here we demonstrate for the first time that human saliva is a suitable sample to detect HCMV-encoded miRNAs.

We assessed five HCMV-encoded miRNAs (miR-UL112-5p, miR-US5-2-3p, miR-UL36, miR-US25-2-3p and miR-UL22A) and addressed whether they are detectable in saliva samples from RTR and whether this marks a high burden of HCMV. HCMV-miRUL112 and HCMV US5-2 have potential human mRNA targets. Although the data are fragmentary, most functions appear to be broadly ‘anti-inflammatory’. This includes targeting genes that reduce the secretion of TNF-α and IL-6 in vitro [17]. HCMV-miRUL22A can be detected in blood from solid organ transplant recipients and interacts in the RNA-induced silencing complex to regulate the expression of several genes [18]. HCMV-US25-2-3p targets *TIMP3,* which is involved in the shedding of the natural killer group 2D (NKG2D) ligand. NKG2D is an activating receptor that promotes cellular cytotoxicity [19]. HCMV-miRUL36 targets the HCMV gene *UL138,* which maintains latency [20]. The miR-US5-2 homolog encoded by RhCMV, miR-Rh183-1, was one of three RhCMV-encoded miRNAs highly expressed in cultured macaque fibroblasts [13].

## 2. Materials and Methods

### 2.1. Study Cohort

Eighty-two RTR were recruited prospectively from renal clinics at the Royal Perth Hospital who met the criteria of clinical stability (>2 years after transplant), no clinical record of HCMV disease or reactivation within 6 months of sample collection, and no current anti-viral treatment. RTR infected with hepatitis B or C were excluded. Eighty-one age and sex matched healthy controls were recruited through local advertisements [6].

Ethics approval was obtained from the Royal Perth Hospital Human Research Ethics Committee (approval number: EC 2012/155) and endorsed by the Curtin University (approval number: HR16/2015). Participants provided written, informed consent [16].

A subset of 32 RTR who had detectable HCMV DNA (in saliva or plasma) or high levels of circulating antibodies were selected to undergo HCMV miRNA detection. These were compared with 12 healthy controls who had high levels of circulating antibodies.

### 2.2. RNA Extraction

Approximately 5 mL of saliva was collected after a water mouth wash by asking the participant to spit into a 50 mL centrifuge tube. Samples were centrifuged for 10 min at 1000× *g*. The saliva pellet and supernatant were separated and stored at −80 °C. Saliva pellets were thawed and mixed with TRI reagent (1:4) before RNA extraction using the MagMAX™ 96 for Microarrays kit (Applied Biosystems, Foster City, CA, USA). RNA extraction was performed with increased isopropanol (125 μL) (Sigma-Aldrich, St Louise, MO, USA) to improve the yield of small RNA molecules, as directed by the manufacturer.

### 2.3. Detection of HCMV-Encoded miRNA

Custom reverse transcription primer pools were generated, and cDNA synthesis for all miRNA assays was performed in a single reaction, according to the manufacturer’s protocols (Applied Biosystems, Foster City, CA. PN 4465407). Pre-designed primer and probe assays targeting mature miRUL112 (assay ID: 469687_mat), miRUS5-2-3p (assay ID: 469255_mat), miRUL36 (assay ID: 006481), miRUS25-2-3p (assay ID: 005400) and miR-UL22A (assay ID: 006040) were sought from Applied Biosystems. RNA from HCMV seronegative healthy participants and uninfected THP-1 cells were used to ensure specificity. These showed no amplification up to 40 cycles. Sensitivity was determined using 10-fold serial dilutions of HCMV AD169 RNA. Samples with cycle thresholds below 10^−4^ dilution of the standard (i.e., after cycle 32–36, depending on the miRNA assayed) were considered negative. All samples were run two to four times and called positive if at least two replicates produced amplification.

### 2.4. Other Assessments of a Persistent HCMV Burden

Detailed methods outlining assessments of HCMV burden have been published [14]. Briefly, plasma IgG titres were assessed using in-house ELISAs based on a lysate of fibroblasts infected with HCMV AD169. Peripheral blood mononuclear cells (PBMC) were used to assess T-cell responses to HCMV lysate and peptide pools derived from IE-1 (JPT Peptide Technologies; Berlin, Germany) via ELISpot assays. We have shown that these antigens stimulate CD4^+^ and CD8^+^ T-cell responses. HCMV DNA was detected in saliva using an in-house qPCR with primers and a probe targeting *UL54*. Samples were considered positive if amplification was achieved before 38 cycles [16]. HCMV DNA was also detected in EDTA plasma using the Abbott Molecular assay (Abbott Laboratories, Chicago, IL, USA) in the Department of Microbiology, Royal Perth Hospital (Western Australia) [21].

### 2.5. Statistical Analyses

Continuous data were analyzed with Mann–Whitney non-parametric statistics, and categorical data were analyzed with Fisher’s exact tests using GraphPad Prism version 8 for Windows (Graphpad Software, La Jolla, CA). *p* < 0.05 is reported as a significant association, but comparisons yielding 0.01 < *p* < 0.05 are noted.

## 3. Results

Thirty-two saliva samples from RTR with detectable HCMV DNA or high levels of circulating antibodies were compared with 12 seropositive healthy controls. There were no significant differences in age, sex or ethnicity between healthy controls and RTR (Table 1).

**Figure 1:**
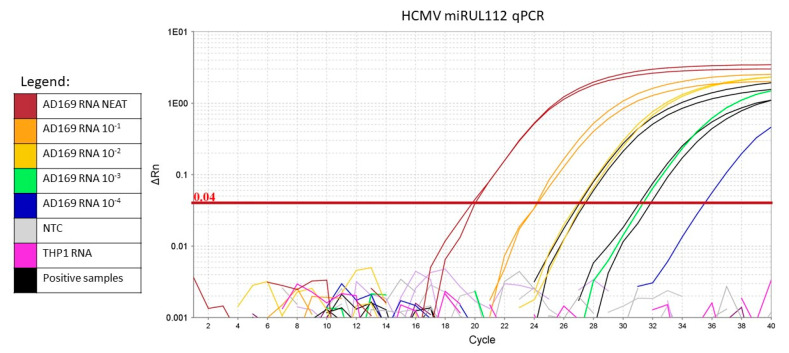
Representative amplification curve from HCMV miR-UL112 qPCR. Coloured curves represent 10-fold dilutions of AD169 RNA. Black curves represent positive samples. No-template control (NTC) and uninfected THP1 RNA are shown in grey and pink, respectively, and do not form curves as no amplification occurred. The horizontal red line represents the threshold.

MiR-US25-2-3p, miR-UL36 and miR-UL112-5p were detected in three, one and two RTR samples (respectively), but not in healthy controls. MiR-UL22a was not detected in any samples. MiR-US5-2-3p was detected in 14 samples from RTR and 3 from controls. In RTR, detection of miR-US5-2-3p was more frequent than any other miRNAs assessed (*p* < 0.0001–0.004). Representative amplification curves generated with a laboratory strain of HCMV (AD169) and clinical samples are presented (Figure 1).

**Figure 2:**
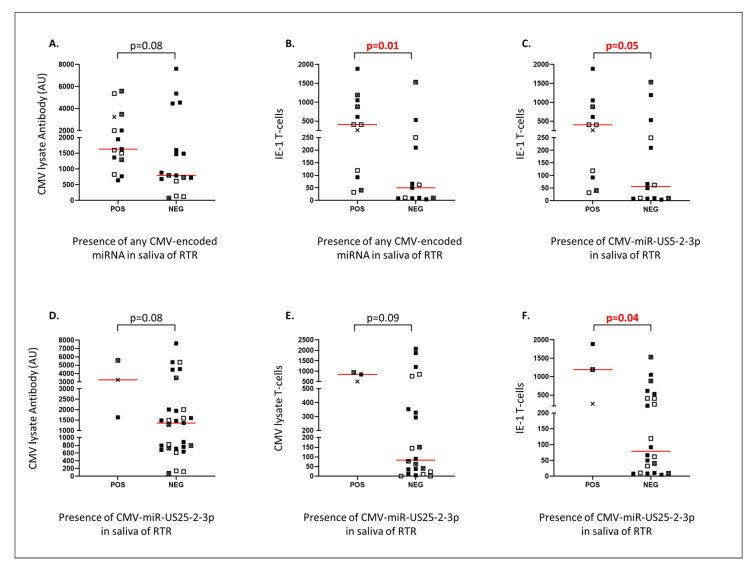
Human cytomegalovirus (HCMV)-encoded microRNAs (miRNAs) were detected using pre-designed assays. Plots (**A**,**B**) compare the levels of HCMV lysate reactive antibodies and IE-1 specific T-cells with the presence of any miRNA assessed. Plot (**C**) compares the levels of IE-1 specific T-cells with the presence of HCMV-miR-US5-2-3p. Plots (**D**–**F**), compare levels of HCMV lysate reactive antibodies and T-cells reactive with HCMV lysate or IE-1 with the presence of HCMV-miR-US25-2-3p. POS: miRNA detected. NEG: miRNA not detected. *p*-values are based on Mann–Whitney tests. ■: HCMV DNA negative, >638 AU of HCMV reactive antibodies. □: HCMV DNA positive in plasma (Abbot assay). ×: HCMV DNA positive in saliva (UL54). ☒: HCMV DNA positive in plasma and saliva.

The presence of any miRNA in saliva from RTR was weakly associated with HCMV lysate antibody levels (Figure 2A, *p* = 0.08) and significantly associated with T-cell responses to HCMV IE-1 (Figure 2B, *p* = 0.01). The association with IE-1-specific T-cell responses remained when miR-US5-2-3p was assessed alone (Figure 2C, *p* = 0.05). Presence of miRUS25-2-3p in saliva of RTR (*n* = 3) also weakly associated with antibody and T-cell responses to HCMV lysate (Figure 2D, *p* = 0.08 and 2E, *p* = 0.09) and associated significantly with increased IE-1 T-cell responses (Figure 2F, *p* = 0.04). There were no associations between the detection of miRNA in saliva and the presence of HCMV DNA in plasma (8/15 with miRNA vs. 6/17 without; Fisher’s exact test, *p* = 0.5) or saliva (5/15 vs. 3/17; *p* = 0.4). Associations with HCMV lysate antibodies and HCMV DNA in saliva were sought in healthy controls, but there were no significant findings (Table S1).

## 4. Discussion

This study provides the first evidence that human saliva contains detectable HCMV-encoded miRNAs, most commonly miR-US5-2-3p. Interestingly the miR-US5-2 homolog encoded by RhCMV, miR-Rh183-1, was highly expressed in cultured macaque fibroblasts [13].

The presence of any HCMV-encoded miRNA, miR-US5-2-3p or miRUS25-2-3p in saliva was associated with increased T-cell responses to HCMV IE-1, rather than with the detection of HCMV DNA. This may reflect the transient expression of HCMV DNA in clinical samples. T-cell responses to HCMV IE-1 have been linked with frequent reactivations of HCMV as IE-1 is the first protein expressed during viral replication, but they build over time and so are elevated in HIV patients stable on antiretroviral therapy (ART) [7]. Our results would be consistent with persistent low-level HCMV replication in the acinar cells of the salivary gland, with episodic outbreaks stimulating systemic responses to HCMV IE-1.

MiR-US5-2-3p may affect the pathobiology of HCMV through interactions with its three known targets-human genes *SNAP23* and *CDC42,* and HCMV gene *US7* [22]. *SNAP23* and *CDC42* are components of the secretory pathway and may affect levels of circulating cytokines [17]. *US7* promotes the degradation of toll-like receptors (TLR) 3 and 4 via ubiquitin-dependent pathways [23]. TLR3 detects dsRNA, which is generated by both RNA and DNA viruses. TLR4 is involved in recognizing viral components, such as envelope glycoproteins and inducing cytokine responses. TLR4 is upregulated in monocytes infected with HCMV and mediates pathways that increase the production of IL-8 and IL-6 [24].

The presence of miR-US25-2-3p in saliva associated significantly with IE-1-specific T-cells and marginally with HCMV lysate reactive antibodies and T-cells (Figure 2E,F). HCMV lysate antigen contains IE-1 protein, so these findings may be related. MiR-US25-2-3p is known to target the human gene *TIMP3*. *TIMP3* may inhibit the process by which an NKG2D ligand, MICA, is shed. NKG2D is expressed on NK cells and CD8^+^ T-cells and increases cytotoxic activity [25]. The reduction in *TIMP3* mRNA activity by miR-US25-2-3p increases the shedding of MICA and, therefore, CD8^+^ T-cell cytotoxicity [19]. This may explain why miRUS25-2-3p was associated with high T-cell responses.

In the cohort presented here, HCMV DNA in saliva was associated with increased T-cell responses to HCMV IE-1 [16], but there was no significant association between the presence of HCMV-encoded miRNA and HCMV DNA. This may reflect the study design favouring samples with HCMV DNA and/or high levels of HCMV-reactive antibody, or differences in the persistence of HCMV DNA and miRNA. However, a study aligning plasma HCMV-encoded miRNAs with HCMV DNAemia in hematopoietic stem cell transplant recipients also found no association [26]. Furthermore, circulating miRNAs are highly stable and can persist packaged into carriers, such as exosome-like particles, whilst retaining the ability to regulate host-gene expression in recipient cells [27]. It is plausible that the persistence of miRNAs in the absence of HCMV DNA may be a stable feature of an individual and may determine the long-term consequences of that infection. This may be determined by the state of “sleepless latency” that has been described in myeloid cells [28]. These questions warrant further investigation.

## 5. Conclusions

Overall, we present preliminary evidence linking detection of HCMV-encoded miRNAs in saliva with altered systemic responses to the virus. Future studies should assess their clinical utility and determine if HCMV-encoded miRNAs persist in other patients with a high burden of HCMV, including neonates and individuals with HIV. Our data establish that these studies could focus on miRUS5-2-3p and miRUS25-2-3p.

## Figures and Tables

**Table 1:** Demographics did not associate with the presence of human cytomegalovirus (HCMV)-encoded microRNAs (miRNAs).

	RTR	Healthy Controls	*p*-Value
*n*	32	12	
**Demographic measures**			
Age (years)	57.5 (31–76)	62 (30–73)	0.27 ^a^
Male/Female	18/14	4/8	0.31 ^b^
Caucasian/Asian/Unknown	26/4/2	10/2/0	0.99
**Presence of HCMV-encoded miRNAs (POS/NEG)**			
HCMV miR-US25-2-3p	3/29	0/12	0.56 ^b^
HCMV miR-UL36	1/31	0/12	0.99
HCMV miR-UL112-5p	2/30	0/12	0.99
HCMV miR-UL22a	0/32	0/12	0.99
HCMV miR-US5-2-3p	16/18	3/9	0.31

^a^ Mann–Whitney test based on data presented as median (range), ^b^ Fisher’s exact test. RTR: renal transplant recipients, POS: positive, NEG: negative.

## Supplementary Materials

The following are available online at https://www.mdpi.com/2311-553X/6/4/50/s1, Table S1: CMV-encoded miRNAs in saliva associate with increased T-cells reactive with CMV IE-1.
