# Supplementary material for: Human Cytomegalovirus-Encoded microRNAs Can Be Found in Saliva Samples from Renal Transplant Recipients
Source: Noncoding RNA. 2020 Dec 18;6(4):50. doi: 10.3390/ncrna6040050 (PMC7768453; doi:10.3390/ncrna6040050)
Supplement: Supplementary file 1 [file ncrna-06-00050-s001.pdf]

Table S1. CMV-encoded miRNAs in saliva associate with increased T-cells reactive with CMV IE-1.

	A	*B*	*A v B*	*C*	*D*	*C v D*
	**RTR with saliva CMV-encoded miRNA**	**RTR without saliva CMV-encoded miRNA**	**P-value**	**Controls with saliva CMV-encoded miRNA**	**Controls without saliva CMV-encoded miRNA**	**P-value[a]**
CMV lysate antibodies (AU)	1633 (638–5582) n = 15	797 (76–7611) n = 17	0.08[a]	482 (472–1496), n = 3	661 (158–1348), n = 9	0.9[a]
IE-1 antibodies (AU)	446 (12–4775) n = 15	173 (5–3646) n = 17	0.4	161 (72–607), n = 3	162 (52–1565), n = 9	>0.9
CMV lysate-specific T-cells[c]	222 (0.5–938), n = 12	90 (0.0–2077) n = 13	>0.9	932 (879–985), n = 2	392 (60–1878), n = 8	Not tested
IE-1-specific T-cells[c]	409 (32–1888), n = 12	50 (5–1533) n = 13	0.01	126 (115–136), n = 2	50 (21–1304), n = 8	Not tested
Presence of CMV DNA in plasma	Positive n = 8, Negative n = 7	Positive n = 6, Negative n = 11	0.5[b]	Not tested	Not tested	
Presence of CMV DNA in saliva	Positive n = 5, Negative n = 10	Positive n = 3, Negative n = 14	0.4	Positive n = 0, Negative n = 3	Positive n = 0, Negative n = 9	Not tested

[a] Mann- Whitney test based on data presented as median (range), [b] Fisher's exact test, [c] expressed as IFNγ spot forming units per 200,000 cells, [d] population induced by CMV and presented as a % of CD3 T-cells.
